# Understanding patient characteristics and medication prescriptions in children with mental health and neurodevelopmental disorders referred to a sleep clinic—A quality improvement/quality assurance analysis

**DOI:** 10.3389/fpsyt.2022.878356

**Published:** 2022-11-10

**Authors:** Osman S. Ipsiroglu, Juhi Bhathella, Renee Paula Boldut, Dean Elbe, Olivia Hill, Elizabeth Keys, Scout McWilliams, Rosalia Silvestri, David F. Wensley

**Affiliations:** ^1^H-Behaviours Research Lab (Previously Sleep/Wake-Behaviours Research Lab), BC Children’s Hospital Research Institute, Vancouver, BC, Canada; ^2^Interdisciplinary Sleep Program, Divisions of Developmental Pediatrics, Respirology, and Child & Adolescent Psychiatry, Departments of Pediatrics and Psychiatry, Faculty of Medicine, The University of British Columbia, Vancouver, BC, Canada; ^3^Healthy Minds Centre, BC Children’s Hospital, Vancouver, BC, Canada; ^4^Department of Pharmacy, Children’s and Women’s Health Centre of British Columbia, Vancouver, BC, Canada; ^5^Department of Psychiatry, Faculty of Medicine, The University of British Columbia, Vancouver, BC, Canada; ^6^Faculty of Health and Social Development, School of Nursing, The University of British Columbia, Kelowna, BC, Canada; ^7^Faculty of Nursing, University of Calgary, Calgary, AB, Canada; ^8^School of Nursing, Dalhousie University, Halifax, NS, Canada; ^9^Centro Interdipartimentale per la Medicina del Sonno UOSD di Neurofisiopatologia e Disordini del Movimento, Messina, Italy; ^10^Dipartimento di Medicina Clinica e Sperimentale, AOU Messina, Messina, Italy; ^11^Interdisciplinary Sleep Program, Division of Respirology, Department of Pediatrics, Faculty of Medicine, The University of British Columbia, Vancouver, BC, Canada

**Keywords:** neurodevelopmental disorders (NDDs), polypharmacy, sleep disorders, mental health, medications, pandemic, waitlist, disruptive behaviour

## Abstract

**Introduction:**

Motivated by challenges faced in outpatient sleep services for mental health and neurodevelopmental disorders (MHNDD) during the COVID-19 clinical shutdown, a pan-Canadian/international working group of clinicians and social scientists developed a concept for capturing challenging sleep and wake behaviours already at the referral stage in the community setting.

**Methods:**

In a quality improvement/quality assurance (QIQA) project, a visual logic model was the framework for identifying the multiple causes and possible interventions for sleep disturbances. Intake forms informed clinicians about situational experiences, goals/concerns, in addition to the questions from the Sleep Disturbances Scale for Children (SDSC), the ADHD Rating Scale-IV and medication history. Descriptive statistics were used to describe the sample.

**Results:**

66% of the pilot study patients (*n* = 41) scored in the SDSC red domains (highest scoring) with highest sub-scores for insomnia (falling asleep 73%; staying asleep: 51%) and daytime somnolence (27%). A total of 90% of patients were taking at least one medication; 59% sleep initiation/sleep medications, 41% in combination with further non-stimulant medications, 9% with stimulants, 27% with antidepressants and 18% with antipsychotics. Polypharmacy was observed in 62% of all patients and in 73% of the ones medicated for sleep disturbances. Qualitative information supported individualisation of assessments.

**Conclusion:**

Our intake process enabled a comprehensive understanding of patients’ sleep and wake profiles prior to assessment, at the referral stage. The high prevalence of insomnia in patients, combined with polypharmacy, requires special attention in the triaging process at the community level.

## Introduction

Over the last decade, an increasing need for sleep assessments and associated services has revealed existing gaps in service delivery. The COVID-19 pandemic has magnified these shortcomings. Outpatient sleep services for children with mental health and neurodevelopmental disorders (MHNDD) is a crucial healthcare domain that needs to be revisited under the paradigm of public shutdowns and exponentially increasing waitlists (1). Up to 80% of this population experience underlying sleep problems, which often remain undiagnosed and untreated (2). The timely and accurate diagnosis of chronic, often familial, sleep disturbances is further hindered by their early onset and overriding disruptive daytime presentations. Early onset and untreated sleep problems aggravate existing daytime presentations and are rarely considered a primary or priority comorbidity, with the familial dimension often being missed (2).

During the COVID-19 clinical shutdown, to overcome existing gaps and proactively react to a rapidly growing waitlist, we created a pan-Canadian working group of clinicians and social scientists and reviewed the applicability of the developed concepts with an international group of sleep researchers. Our group reviewed the challenges faced in outpatient paediatric sleep services and agreed on the need for a set of “universal” first line interventions for sleep disturbances that could be utilised in community-based settings. These first line measures were reviewed by international members of the group (3, 4). As causes of sleep disturbances can be diverse and complex, the team first agreed to develop a visual logic model for capturing the possible causes of common paediatric sleep disturbances and mapped these to potential “first-line” intervention options. The quality improvement/quality assurance (QIQA) protocol was developed with the aim of identifying potential risk factors that could be targeted at the referral level in family medicine, paediatrics, and child and adolescent psychiatry. The current version of the QIQA protocol suggests a structured intake process utilising a mixed methods approach using open-ended questions (5), including individual goals (6) and concerns (7) of the patient/family with regards to sleep and daytime functioning, medication information, and validated questionnaires for capturing both sleep and wake behaviours (8, 9). The newly developed intake forms were tested in an ambulatory one-to-one service delivery setting for children and adolescents with MHNDD at an academic sleep programme. The goal of this brief report is to describe the trends of patient characteristics in a pilot cohort at the time of referral to the sleep programme, all assessed using this QIQA protocol.

## Methods

### Time and location of the quality improvement/quality assurance project

The project started in May 2020 as a pan-Canadian endeavour. Eight scientists (health management/decision support) and five parent advocates joined the pan-Canadian working group consisting of 19 clinicians (7 community-based/12 working in an academic environment). Canadian group members were located in non-hospital and/or research settings in urban (Vancouver, Regina, Winnipeg, Moncton), rural and remote locations (Rexton, Rogersville, Saint-Louis de Kent). International group members, who joined the endeavour over 2020, were located in academic settings in Australia, Austria, Germany, United Kingdom, and Italy. Patient data collection started at the first project site (Vancouver, Canada) in September 2020; the first pilot data were collected at the Interdisciplinary Sleep Clinic of BC Children’s Hospital between September-November 2020; the project is currently ongoing and data are collected electronically with REDCap, an electronic data collection tool (10). Under instruction, student research assistants developed the electronic database in REDCap format and performed the analysis; the backend is available for other clinics. International working group members contributed as peer reviewers to this discourse.

### Ethics approval

The BC Children’s Hospital based QIQA project was registered with the Provincial Health Service Authority, PHSA, British Columbia and electronic data collection approved by the institutional Clinical Research Informatics Committee—a joint committee of PHSA and Research Ethics Board at the University of British Columbia.

### The logic model

**FIGURE 1:**
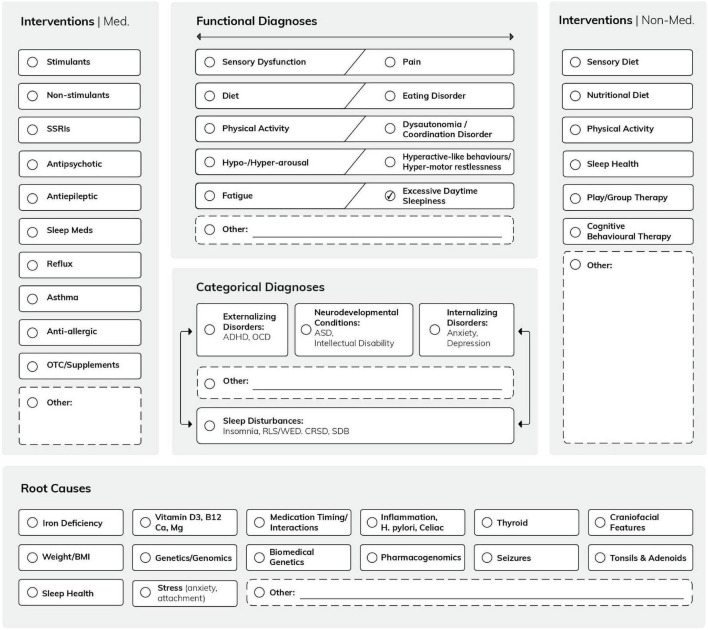
The *Mind-the-Gap* logic model shows the interconnections of several contributing factors (“possible root causes”) with functional and categorical diagnoses. This conceptual framework allows the addition of further functional diagnoses, possible causes, and interventions, as it is designed as a grid. (Note that the logic model is a conceptual framework and its applicability is tested in clinical practice) (49).

The logic model is based on the working group discussions about how paediatric sleep disturbances could be assessed and managed in a community setting and supported by developmental paediatrics and mental health clinics. Both of these clinical settings have implemented transdisciplinary and transdiagnostic approaches. The first task for the working group was to use their clinical, social science and parenting expertise to review to what degree sleep is related to functional diagnoses and/or root causes—all factors, which are often not recognised when recommending first-line interventions in the community setting. The model positions sleep in the centre, which encourages the clinical consideration of sleep disturbance as a possible comorbidity, underlying, and/or aggravating factor of any developmental and/or mental health condition. Further, this empirical logic model allows clinical team members not only to review sleep problems within the context of categorical and/or functional diagnoses that drive clinical practice, but also to review the possible interventions and discuss with the patients their priorities (3, 4). The visual representation of the logic model is depicted in Figure 1.

### Pilot project

This pilot project was performed as part of an ongoing QIQA project with data collected over a period of 3 months, between September-November 2020 at the BCCH Interdisciplinary Sleep Program in Vancouver, BC.

### Intake questionnaire

All patients referred for a sleep assessment received an intake questionnaire, which consisted of open-ended questions (5), including individual goals (6) and concerns (7) of the patient/family with regards to sleep and daytime functioning, medication information, and the Sleep Disturbances Scale for Children (SDSC) (8). After the first intake, as attention-deficit hyperactivity disorder (ADHD) was identified as a main diagnosis, we also implemented the (open source) ADHD Rating Scale-IV for capturing the interconnection between sleep and wake behaviours (9).

The questionnaire was applied during the pilot testing period of 3 months in downloadable and/or clickable PDF format sent by email. With an initial phone call, the service booking clerk informed the patients/caregivers about the procedure and those who consented sent back the completed forms.

The SDSC was used for describing the type and severity of sleep problems (8). The SDSC is a 26 question, parent-reported scale that is capable of distinguishing six classes of sleep disturbance: disorders of initiating and maintaining sleep (DIMS), sleep disordered breathing (SDB), sleep wake transition disorders (SWTD), disorders of excessive somnolence (DOES), sleep hyperhidrosis (SHY) and disorders of arousal (DA) (8, 11). Scores were grouped into five different colour groups (dark red, red, amber, yellow, green), with dark red signifying the highest (most severe) score and green signifying the lowest (least severe) score.

Medications were grouped into the following categories: (1) medications for sleep initiation/sleep (i.e., melatonin, zolpidem), (2) benzodiazepines, (3) stimulants, (4) non-stimulants (i.e., clonidine, guanfacine, atomoxetine), (5) antidepressants, (6) antipsychotics, (7) anti-epileptics, and others (e.g., antidiabetic medications, thyroid, and growth hormones). Paediatric polypharmacy was defined as ≥ 2 concurrent medications for ≥ 1 day (12).

The ADHD Rating Scale-IV is an 18-item easy-to-administer questionnaire for diagnosing ADHD in children and adolescents and following up treatment success similar to the SDSC. We utilised the parent questionnaire pertaining to home behaviours in English (9).

Goals (6) and concerns (7) of the patient/family were categorised using the BEARS concept (5). The BEARS mnemonic (B for bedtime, E for excessive daytime sleepiness, A for awakenings, R for routines, S for snoring or sleep disordered breathing) and two additional categories were added in our analysis to capture the quality of sleep (Q) concerning events such as sleep hyperhidrosis, enuresis, and/or night binge eating; the other being goals or concerns not specific (NS) to the patient’s sleep (e.g., less medication, or parents wanting to sleep better themselves).

## Results

### Pilot project data

During the 3-month pilot period, we received 51 referrals. Fifty-one families were contacted, 41 returned a completed intake form (response rate 80%). Those who did not respond received an invitation to fill out the forms under the guidance of a professional from the clinic. Here, we are presenting the data of these 41 consecutive patients between the ages of 3 and 18, who were seen between September and November 2020, who all were eligible for the sub-specialty sleep/wake-behaviours clinic. Patient characteristics are presented in Table 1.

**TABLE 1:** Patient characteristics of 41 consecutive patients between the ages of 3 and 18, who were seen between September and November 2020.

Demographics of the patient cohort (*n* = 41, mean 11.3 years, median 11y, min 3y, max 18y)	No. of patients with confirmed diagnosis, n
**Neurodevelopmental conditions**	
Autism spectrum disorder (ASD)	12
Foetal alcohol spectrum disorder (alcohol related neurodevelopmental disorder; *in utero* exposure)	6 (4; 2)
Global development delay and intellectual disability (mild to severe)	12
Genetic conditions (Down syndrome; Prader-Willi Syndrome; Trisomy 13 Mosaic; Trisomy X; Noonan syndrome)	6 (2; 1; 1; 1; 1)
Neurologic conditions [motor disorders (Cerebral Palsy; Leigh Syndrome); epilepsy, visual impairment; septo-optic dysplasia; mild traumatic brain injury]	10 (1; 1; 2; 2; 1; 3)
Sensory processing dysfunctions	29
Others (tics; hypothyroidism; chronic headaches; Type 1 Diabetes)	6 (1; 3; 1; 1)
Self-injurious behaviours/suicidal ideation	8 (6; 2)
**Mental health diagnoses/comorbidities**	
Externalising disorders or disorders of disruptive challenging behaviours	
ADHD	23
Oppositional defiant disorder	3
Obsessive compulsive disorder	1
Internalising disorders	
Anxiety disorders	20
Emotional dysregulation (depression; mood disorders, including dysthymia)	11 (8; 3)
Bipolar disorder	1
**Sleep disorders (working diagnoses)**	
Insomnia	40
Excessive daytime sleepiness	37
Circadian rhythm sleep disorder (CRSD; delayed sleep onset; polyphasic patterns)	32 (31; 1)
Parasomnias	31
Sleep-disordered breathing	22
Probable/possible RLS implicating necessity for structured behavioural observations and blood work investigations (e.g., iron deficiency)	37

Note that multiple conditions may apply for one individual.

Based on the available information by the referring provider, intake forms and/or available information from existing hospital charts, 8/41 (20%) patients had a dual diagnosis of ADHD and ASD, 15/41 (37%) had ADHD alone and 4/41 (10%) had ASD alone. Other diagnoses included anxiety (20/41, 49%), global developmental delay/intellectual disability (12/41; 29%), depression (8/41; 20%); foetal alcohol spectrum disorder (FASD/*in utero* exposure 6/41), and self-injurious behaviours (SIB; 6/41) both 15%.

Two-thirds of the patients 27/41 (66%) scored in the red domain of SDSC (either dark or light red; for the purposes of this descriptive paper, we have collapsed the dark red and red groups together). For the subscale scores, 30/41 (73%) of patients had scores in the red domain for DIMS; 3/41 (7%) for SDB concerns; 10/41 (24%) for DA; 21/41 (51%) for SWTD; 11/41 (27%) for DOES; and 5/41 (12%) for SHY. As the ADHD Rating Scale was added to the intake forms later on, only 13 participants filled it out. 10/13 (77%) scored red overall in their age adjusted percentiles.

37/41 (90%) patients were prescribed at least one medication. As North American regulations differ from European ones, using international terminology, we listed melatonin as a sleep medication and not as an over the counter (OTC) drug. 22/37 (59%) were taking sleep initiation/sleep medications (one patient was taking melatonin and zolpidem); 9/22 (41%) were taking these agents in combination with further non-stimulant medications (i.e., clonidine, guanfacine and atomoxetine), 6/22 (27%) with antidepressants (e.g., fluoxetine, fluvoxamine, trazodone, sertraline, escitalopram), of these six patients, four were also on antipsychotics (e.g., quetiapine, risperidone). In addition, sleep initiation/sleep medications were used in combination with stimulants in 2/22 (9%). Stimulants were prescribed seven times within the entire cohort, four times in combination with non-stimulants, three times with antidepressants and one time with an antipsychotic. Within the group of 22 patients treated for sleep disturbances, polypharmacy was seen in 16/22 (73%) cases, within the group of 37 medicated patients in 23/37 (62%) cases, and within the entire patient cohort in 23/41 cases (56%).

**FIGURE 2:**
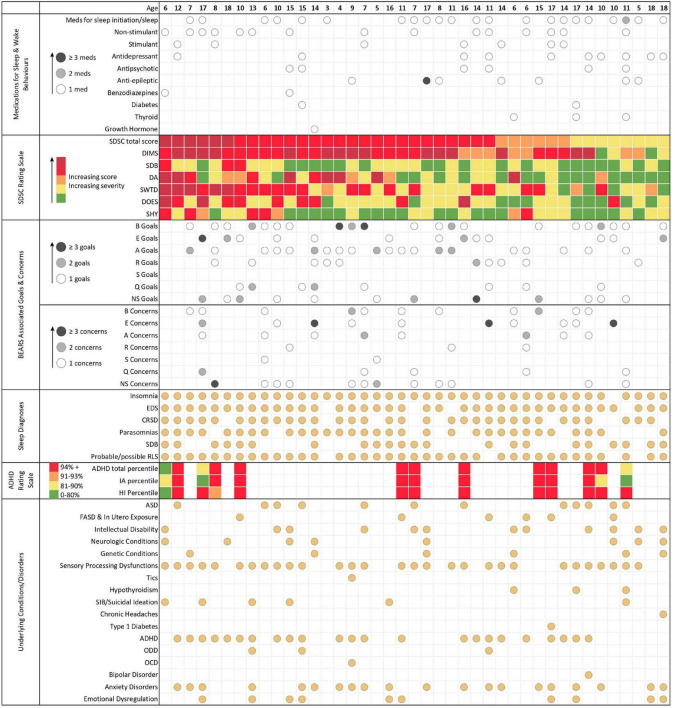
An overview of intake data. Each column represents the data for one individual patient. Patients are arranged according to SDSC total score (highest scores from the left. From the top to the bottom: MEDICATION DATA. Medications have been grouped according to categories (e.g., sleep initiation/sleep; non-stimulant, etc.). One medication is marked with a white circle, two medications are marked with a grey circle, and three or more medications are marked with a black circle. Note that melatonin (using international terminology) is listed as a medication and not as an over the counter drug. SDSC TOTAL SCORES AND SUBSCORES. DIMS, disorders of initiating and maintaining sleep; SDB, sleep disordered breathing; DA, disorders of arousal; SWTD, sleep-wake transition disorders; DOES, disorders of excessive somnolence; SHY, sleep hyperhidrosis. Each coloured dot represents a different category of SDSC scoring that increases (and severity of symptoms) from green (lowest score), to yellow, to amber, to red, and finally dark red (highest score). GOALS/CONCERNS. Information on goals and concerns of the patients has been grouped using the BEARS themes. SLEEP DIAGNOSES. The sleep medicine working diagnoses, which were made after the first assessment, are presented in the lowest block. EDS, excessive daytime sleepiness; CRSD, circadian rhythm sleep disorder; SDB, sleep disordered breathing; RLS, restless legs syndrome. ADHD Scores. IA, inattention and HI, hyperactivity-impulsivity sub-scores. Note that ADHD scoring was only available in 13 patients.

Figure 2 shows the patients (*n* = 41, mean 11.3 years, median 11y, min 3y, max 18y) arranged according to SDSC total scores (highest scores from the left) in the context of prescribed medications and patient/parent goals and concerns. The youngest patient taking melatonin as a sleep medication was 3 years of age; a patient 6 years of age was taking two medications for sleep and/or wake behaviours (clonidine, melatonin); a boy 11 years of age, requiring complex chronic care management, was taking six plus medications for sleep [clonidine, melatonin, zolpidem, gabapentin, quetiapine, trazodone, plus prescribed cannabidiol, and THC (tetrahydrocannabinol)] in addition to synthroid. The youngest patient taking an antidepressant was 10 years of age, whilst the youngest patient taking an antipsychotic was 11 years of age. The patient (6 years of age), who scored dark red/red in the SDSC total score and all subscores was taking a benzodiazepine for epilepsy treatment (clobazam) and non-stimulant (clonidine), both given in the evening for improving sleep.

Goals and concerns were not available for 5 patients; including the two with the highest SDSC scores, two with red scores in the DIMS domain and one with the second lowest scores; in these cases, clinical assessment revealed that non-restorative sleep (sleep quality) was an issue of concern. We grouped family goals and concerns according to the domains in the BEARS themes. Insomnia (B + A: 30/36) was the main concern with 83%. In detail, *B* for: bedtime/falling asleep related comments (24/36; 67%); *E* for excessive daytime sleepiness, also low energy, naps, and trouble waking up in the morning (18/36; 50%); *A* for awakenings and sleep maintenance but also trouble falling back asleep, nightmares, and parasomnia in general (e.g., sleep walking, talking) (22/36; 61%); *R* for routines, regular duration, regular bed and waketime, and getting enough sleep (12/36; 33%); *S* for snoring and sleep disordered breathing (2/36; 6%). We included all comments on quality of sleep in the *Q* theme, which were otherwise not captured, such as sweating, bed wetting or night binge-eating (11/36; 31%). Finally, *NS* stands for not specific to the participant’s sleep (19/36; 53%). Examples of goals that highlight the specific hopes and worries of families are: *B*, “we would like him to go to bed without anger”; *E*, “Get out of bed without feeling extremely tired independent of sleep amount”; *A*, “To not wake up screaming at night”; *R*, “Sleep the typical number of hours for his age”; *S*, *N/A* in this patient cohort; *Q*, “helping him feel like he has had a restful sleep”; Non-sleep specific goals, “emotional regulation.” Exemplar patient (family) reported concerns were: *B*, “He needs to be touching or feeling someone beside him or he will not sleep”; *E*, “Chewing gum and exercise breaks needed to stay awake during classes”; *A*, “Long term effects of sleep interruption”; *R*, “He does not get enough sleep at night time.” *S*, “that she has sleep apnea, again.” *Q*, “she is not getting a good sleep”; non-sleep specific concerns, “that she will not be developing to her appropriate age.”

The initial working diagnoses (after clinical assessment) from a sleep medicine perspective are shown in the second part of Table 1. Note that the majority of the patients (40/41; 98%) were suffering from insomnia, fulfilling the criteria for circadian rhythm sleep disorders (CRSD; 32/41; 78%; mainly delayed sleep onset 31/32) and affecting daytime with excessive daytime sleepiness or affected working speed (37/41; 90%). Parasomnias (31/41; 76%) and SDB (22/41; 54%) followed in the ranking. With the high amount of comorbid sensory processing dysfunction (29/41; 71%), probable or possible restless legs syndrome (RLS) requiring structured behavioural observations (13) and haematological and/or functional iron deficiency investigations (37/41; 90%). Probable or possible RLS was considered a main possible organic cause and became a frequent diagnosis.

## Discussion

This joint community academia collaboration resulted in *three* major achievements. *First*, the development of a context-framing logic model to capture the multiple causes and intervention options for sleep disturbances; *second*, the development and application of a mixed methods approach for a structured intake process for complex patients with MHNDD; and *third*, a clinical phenotyping of a pilot cohort using this mixed methods approach.

### The logic model

Our logic model framed not only the entire QIQA project but also the assessment in each individual case. We called our logic model “Mind-the-Gap” to emphasise its added value in identifying otherwise poorly captured information related to the complexity of sleep disorders in children and adolescents, which, unrecognised, may initiate a cascade of mental health diagnoses (14, 15). An example for a “mind-the-gap” alert are functional diagnoses (e.g., sensory dysfunctions), usually observed by parents/caregivers and/or allied health care professionals, but not necessarily flagged, or identified by physicians in the community (16). As such, the interconnections of sensory dysfunctions with categorical day and nighttime-related diagnoses are not always clear to the providers who make the decisions for further investigations or interventions. Similarly, the interconnections between the wide range of functional diagnoses and potential root causes are often now recognised. Typical examples of root causes of sleep and challenging daytime behaviours are biochemical imbalances, such as iron (17, 18) or vitamin D deficiencies (19, 20).

### Clinical phenotyping

Intake information and subsequent clinical assessment resulted in insomnia diagnosis for 98% of all cases. Among the goals and concerns, insomnia (83%) and affected daytime behaviours/DOES/EDS (50%) were explicitly expressed as main concerns. Note that 53% had NS related goals and concerns, revealing that parents connected disturbed sleep with affected daytime behaviours. CRSD-like presentations and parasomnias were diagnosed in 78 and 76% of the cases with insomnia. In 90% of our pilot cohort, probable RLS, requiring further structured behavioural observations (13) and blood work investigations (21), was likely contributing to insomnia and disturbing sleep. The high number of possible RLS diagnoses requiring further investigations may be due to the specific referral pathways qualifying for the specific developmental paediatrics/child and adolescent psychiatry associated *behavioural sleep medicine* and not for respiratory or neurology associated sub-speciality sleep medicine clinics. The rationales for daytime related referral diagnoses were (from a sleep medicine perspective) partly difficult to understand, as it was not obvious who initially had established the diagnosis (e.g., ADHD and/or other mental health diagnoses), under which circumstances the diagnosis was established (e.g., whether sleep problems had been excluded or not) and what measures had been applied to alleviate symptoms (e.g., trials for sleep health recommendations) (22, 23). The low number of patients in this pilot project precludes our ability to assess correlations between sleep disorders, mental health diagnosis and other comorbidities. Continuing to use the logic model to frame clinical presentations will result in larger patient cohorts that will allow us to phenotype patients referred to our clinic with ADHD, MHNDD and sleep disturbances further, as recently suggested (24, 25).

### Medication characteristics

Fifty-nine percent of patients were taking medications targeting insomnia (i.e., melatonin and zopiclone), 41% were taking these agents in combination with further non-stimulant medications (i.e., clonidine, guanfacine, atomoxetine), 9% with stimulants, 27% with antidepressants, and 18% with antipsychotics. While stimulants and non-stimulants can cause insomnia (18, 26), antidepressants and antipsychotics can negatively affect sleep quality and architecture (27, 28). Note that in Canada, most psychiatric medications have a regulatory status of non-approval for treatment of children and adolescents (with the exception of ADHD treatments). Such medication use may still be rational and evidence-based (for example, use of risperidone and aripiprazole for treatment of irritability in autism) (29). However, while polypharmacy may be necessary in a patient with a MHNDD and comorbidities (30), the fact that patients required a referral to sleep medicine despite the medications trials, raises the concern to what degree sleep, as a complex neurophysiologic function, had been integrated in previous assessments and choice of treatment strategies. The youngest patient taking sleep medication (melatonin) was 3 years of age, an age for which the use of behavioural strategies to manage difficulties with sleep initiation and maintenance are highly effective (31). Further, in the 13 referrals where ADHD ratings were assessed, 10/13 patients scored red in the ADHD and in one or more SDSC domains. Overall high percentage of patients scoring red in the DIMS, SWTD and DOES domains of the SDSC and who received clinically affected daytime behaviours or EDS as a working diagnosis supports the notion that sleep was not sufficiently assessed and/or treated. It is possible that the COVID-19 related shutdown in 2020 had an enhancing effect on prescription practices and on the incidence of polypharmacy within this population; however, as we have not analysed any medication data prior to the onset of COVID-19, we can only speculate on this aspect, which is an important limitation of our data.

### The role of over the counter drugs

Not only does this QIQA project allow for the ongoing analysis of medication data, it also reveals an eye-opening insight to medication practices and the necessity for medication reconciliation for outpatient clinics on an ongoing basis. Patients’ goals and concerns may support this process and allow a more patient-oriented perspective within the framework of the logic model. While PharmaNet, a network linking all pharmacies within the province of British Columbia to a central set of data, captures information from every outpatient prescription dispensed in British Columbia, OTC drugs that may have been used for sleep (e.g., melatonin, antihistamines) are not captured. The main *“sleep medication”* not captured is melatonin, regulated as a prescription drug all over the world, except in North America. Melatonin is an internal cue hormone that synchronises the organism’s biological rhythms and indoleamine, adjusting circadian rhythmicity (32). It should be noted that melatonin is *not* a sedative, therefore, should not be used as such (32–34). High dosage applications of melatonin for improving sleep maintenance are obsolete (35) and can pave the way for further medications, as shown in a subgroup of patients with FASD (36). We see it as concerning that the majority of these children received sleep and sedating medication in combination with other prescription drugs, yet the insomnia symptoms continue to be unresolved, likely resulting in the aggravation of behavioural daytime challenges due to non-restorative sleep.

### Prescription drugs and first line measures

Among the numerous prescription and non-prescription medications used for sleep, only a few have been investigated in high quality trials, systematic and scoping reviews, and meta-analyses for the paediatric population. Interestingly, most medications used for insomnia, such as clonidine, are used off-label. Clonidine is a non-selective alpha agonist that reduces sympathetic outflow from the central nervous system, causing a decrease in arterial blood pressure and wakefulness (37). Medications such as antidepressants and antipsychotics are commonly employed off-label to treat sleep disturbances, despite limited evidence (38, 39). In contrast, the non-pharmacological intervention of correcting biochemical imbalances, such as iron and vitamin D deficiencies, as an effective management of DIMS and SWTD (e.g., insomnia), has been investigated in multiple trials, systematic and scoping reviews, and meta-analyses (17–20). Similarly, in the domain of SDB, corticosteroid nasal sprays have been implemented as a conservative first line treatment before surgical interventions and/or continuous positive airway pressure (CPAP) are considered (40, 41).

The critical discourse regarding how to use psychotropic medications in children with MHNDDs has been ongoing for several years (42–44). Trends to medicate children seem to be specific to North America. Provincial PharmaNet data has shown that the percentage of children who received psychotropic drugs was two-to-five times higher than rates reported in European countries (45). However, it is worth noting that these rates, although high, are still 30% lower than rates reported in the US (45). Most recently, in the symposium at the Canadian Sleep Society (CSS) 2021 meeting, Bruce Carleton, who had analysed the B.C. PharmaNet data, showed that 35,870/145,170 (24.7%) children under age 5 have been dispensed psychotropic drugs (over the time period 1997–2017; (46). Further, 2,125 children under the age of five had used stimulants; interestingly, only 52.3% (1,112/2,125) of these children had received a diagnosis of ADHD before the prescription was made, implying that the other 47.7% had not yet received a diagnosis of ADHD before commencing stimulants.

## Conclusion

The analysis of this pilot project with a limited number of patients reveals insomnia, affected daytime wellbeing/EDS and polypharmacy in the majority of the cases. We know that individuals with MHNDD are at high risk for psychotropic medications (47) and our current understanding is that unrecognised sleep disturbances can often aggravate mental health manifestations and have negative consequences on family dynamics and coping skills (48). In the context of this QIQA project, we have tried to explore pharmacologic interventions in children with MHNDD with qualitative and questionnaire based quantitative data at the entry point to the clinical sleep service. (3, 4). Our findings, despite reporting only a small patient cohort, suggest that chronic insomnia symptoms of patients with MHNDD are not being successfully treated. The electronic forms, which have been developed within this QIQA project, will allow the creation of an electronic registry (10), which may shed light on this challenging and controversially discussed topic with a larger number of patients.

## Data availability statement

The original contributions presented in this study are included in the article/supplementary material, further inquiries can be directed to the corresponding author.

## Ethics statement

The BC Children’s Hospital based QIQA project was registered with the Provincial Health Service Authority, PHSA, British Columbia and electronic data collection approved by the institutional Clinical Research Informatics Committee—a joint committee of PHSA and Research Ethics Board at the University of British Columbia. Written informed consent to participate in this study was provided by the participants’ legal guardian/next of kin.

## Members of the international and Canadian working groups

“Virtual Home Visits Addressing Needs of Waitlisted Vulnerable Paediatric Patients - Learning Lessons from the Pandemic Shutdown/Visites virtuelles à domicile en réponse aux besoins des patients pédiatriques vulnérables en attente de services - tirer des leçons de la pandémie” who contributed to protocol development: Katie Allen (Department of Psychiatry, UBC), Anthony Bailey (Department of Psychiatry, UBC), Nadia Beyzaei (BCCH Research Institute, UBC), Sarah Blunden (Appleton Institute of Behavioural Science, Australia), Bruce Carleton (Department of Pediatrics, UBC), Elizabeth Cooper (Faculty of Kinesiology & Health Studies, University of Regina), Georg Dorffner (Center for Medical Statistics, Informatics and Intelligent Systems, Medical University of Vienna, Austria), Dean Elbe (Department of Pharmacy, Children’s and Women’s Health Centre of British Columbia), Robin Friedlander (Department of Psychiatry, UBC), Sarah Gander (Department of Pediatrics Horizon Health Network), Denise Gabriel (Faculty of Science, UBC), Mary Glasgow-Brown (School of Occupational Science and Occupational Therapy, UBC), Janet Greenman (Department of Pediatrics, UBC), Osman Ipsiroglu (Department of Pediatrics, UBC), Elizabeth Keys (School of Nursing, UBCO), Gerhard Kloesch (Department of Neurology, Medical University of Vienna, Austria), Calvin Kuo (Department of Biomedical Engineering, UBC), Mansfield Mela (Department of Psychiatry, University of Saskatchewan), Onawa Labelle (Department of Psychology, University of Windsor), Nicole LeBlanc (Vitalité Health Network, New Brunswick), Suzanne Lewis (Department of Medical Genetics, UBC; Pacific Autism Family Network), Christine Loock (Department of Pediatrics, UBC), Susan McCabe (School of Medical and Health Sciences, Edith Cowan University, Perth, Australia), Jacqueline Pei (Department of Educational Psychology, University of Alberta), Anamaria Richardson (Department of Pediatrics, UBC), Dorothee Reid (Family Advisory Committee, Canadian FASD Research Network), Rosalia Silvestri (Department of Clinical & Experimental Medicine, Messina University, Italy), Sylvia Stockler (Department of Pediatrics, UBC), Lori S. Vitale Cox (Eastern Door Centre, Elsipogtog, New Brunswick), David Wensley (Department of Pediatrics, UBC), Thomas Wetter (Department of Psychiatry and Psychotherapy, University of Regensburg), Luci Wiggs (Oxford Brookes University Oxford, United Kingdom).

## Author contributions

OI developed the logic model, QIQA project, and wrote the manuscript. RS was involved in the development and international peer review of the logic model. EK and DW helped with implementation of the QIQA project and edited the manuscript. JB and RB developed the electronic intake forms using REDCap and were responsible for backend management of the project. JB, RB, SM, and OH carried out data analysis. SM and OH created all graphics and helped edit the manuscript. DE provided in-depth input regarding pharmacological questions and helped edit the manuscript. All authors contributed to the article and approved the submitted version.

## Abbreviations

ADHD: attention-deficit/hyperactivity disorder
ASD: autism spectrum disorder
BEARS: concept or themes
B: for bedtime
E: for excessive daytime sleepiness
A: for awakenings
R: for routines
S: for snoring or sleep disordered breathing
Q: for quality of sleep
NS: not specific to the participant’s sleep
CRSD: circadian rhythm sleep disorder
EDS: excessive daytime sleepiness
FASD: foetal alcohol spectrum disorder
MHNDD: mental health and neurodevelopmental disorders
OCD: obsessive compulsive disorder
ODD: oppositional defiant disorder
OTC drugs: over the counter or non-prescription drugs
QIQA: quality improvement/quality assurance
RLS: restless legs syndrome
SDSC: sleep disturbances scale for children
DIMS: disorders of initiating and maintaining sleep
SDB: sleep disordered breathing
SWTD: sleep wake transition disorders
DA: disorders of arousal (DA)
DOES: disorders of excessive somnolence
SHY: sleep hyperhidrosis
SIB: self-injurious behaviours.
